# Microexpressions Differentiate Truths From Lies About Future Malicious Intent

**DOI:** 10.3389/fpsyg.2018.02545

**Published:** 2018-12-18

**Authors:** David Matsumoto, Hyisung C. Hwang

**Affiliations:** ^1^Department of Psychology, San Francisco State University, San Francisco, CA, United States; ^2^Humintell, El Cerrito, CA, United States

**Keywords:** microexpressions, facial expressions of emotion, veracity, deception, checkpoints

## Abstract

The few previous studies testing whether or not microexpressions are indicators of deception have produced equivocal findings, which may have resulted from restrictive operationalizations of microexpression duration. In this study, facial expressions of emotion produced by community participants in an initial screening interview in a mock crime experiment were coded for occurrence and duration. Various expression durations were tested concerning whether they differentiated between truthtellers and liars concerning their intent to commit a malicious act in the future. We operationalized microexpressions as expressions occurring less than the duration of spontaneously occurring, non-concealed, non-repressed facial expressions of emotion based on empirically documented findings, that is ≤0.50 s, and then more systematically ≤0.40, ≤0.30, and ≤0.20 s. We also compared expressions occurring between 0.50 and 6.00 s and all expressions ≤6.00 s. Microexpressions of negative emotions occurring ≤0.40 and ≤0.50 s differentiated truthtellers and liars. Expressions of negative emotions occurring ≤6.00 s also differentiated truthtellers from liars but this finding did not survive when expressions ≤1.00 s were filtered from the data. These findings provided the first systematic evidence for the existence of microexpressions at various durations and their possible ability to differentiate truthtellers from liars about their intent to commit an act of malfeasance in the future.

## Introduction

Microfacial expressions of emotion (hereafter microexpressions) have been considered a reliable indicator of deception for decades because of the influence of books (e.g., Ekman, 1985, 2009) and mass media. Despite their decades-long influence and the many claims that have been made about them, however, especially about their duration, there have been surprisingly few studies that have actually measured different expression durations and tested which, if any, differentiate truths from lies. This exploratory study addresses this issue. We begin first with a critical review of the concept and claims about microexpressions, including a discussion of what microexpressions are and the possible ways of operationalizing them.

### A Critical Review of the Concept of Microexpressions

#### Origins

Haggard and Isaacs (1966) were the first to discover microexpressions over half a century ago in their review of films of psychotherapy sessions. They wrote:

“We first noticed the existence of micromomentary expressions (MMEs) while scanning motion picture films of psychotherapy hours, searching for indications of non-verbal communication between the therapist and patient. Although we discovered something new each time we ran the film at normal speed, we also found it instructive to run the film silently backward or forward, faster or slower, or even frame-by-frame. In fact, such procedures often seemed to give a more vivid picture of the nonverbal aspects of the therapist–patient interchange than when the film provided a reproduction of the events at the same speed with which they transpired in the therapy. During such explorations, for instance, we noted that occasionally the expression on the patient’s face would change dramatically within three to five frames of film (as from smile to grimace to smile), which is equivalent to a period of from one-eighth to one-fifth of a second. We were not able to see these expression changes at the normal rate of 24 frames per second (f.p.s.). Being intrigued by this phenomenon, we set out on a long and tedious attempt to study the occurrence and meaning of these micromomentary expression changes and to relate them to other aspects of the therapeutic process.” (Haggard and Isaacs, 1966, p. 154).

Prefacing the notion that microexpressions were possible indicators of deception, Haggard and Isaacs (1966) analyzed the relation between microexpressions and the manifest verbal content spoken when microexpressions occurred and suggested that inconsistencies between them were areas of concealed thoughts and feelings (see Table 2, Haggard and Isaacs, 1966, for a summary of their findings, p. 162).

The idea that microexpressions exist is rooted in Darwin’s (1872) inhibition hypothesis, which suggested that facial actions that cannot be controlled voluntarily may be produced involuntarily when individuals try to suppress their expressions. The neuroanatomical bases of emotional expressions suggest how this occurs: Facial expressions are under the neural control of two distinct areas of the brain, one controlling voluntary movements and the other controlling spontaneously occurring, involuntary movements (Rinn, 1984, 1991; Matsumoto and Lee, 1993). When individuals are emotional but need to control their expressions, both systems engage in a neural “tug of war” over control of the face, allowing for quick, fleeting leakage of expressions, which are microexpressions. Haggard and Isaacs (1966) referred to this process as “temporal censorship” (although they did not refer to Darwin’s work).

After their initial discovery, attention to microexpressions as possible clues to hidden emotions and deception increased primarily through the writings of Ekman and Friesen, who first wrote about them in two book chapters that described their review of interviews with psychotherapy patients (Ekman and Friesen, 1969, 1974). Subsequently, microexpressions were made popular in descriptions in trade books about emotions and deception (Ekman, 1985, 2003, 2009). In these works, microexpressions were usually defined as fleeting emotional expressions lasting between 1/25th to 1/5th s (i.e., 0.04–0.20 s), not noticeable to the naked eye, and expressed involuntarily. Even today, a major proponent of microexpressions defines them even more restrictively as “facial expressions that occur *within* 1/25th of a second” (emphasis ours; Micro Expressions, 2018; “What are microexpressions?,” 4 September).

#### Previous Research

Despite their contributions and popularity as possible indicators of deception, however, until recently no production study published in a peer-reviewed journal had documented their existence when individuals are lying. (A production study is one in which emotions are elicited in individuals and the corresponding facial expressions that are produced are measured.) But several recent studies have remedied this gap. Porter and ten Brinke (2008) coded facial expressions of emotion that were produced by participants who viewed emotional images and responded to each with a genuine or deceptive expression and reported that microexpressions were exhibited by only 21.95% of participants in 2% of all expressions. ten Brinke and Porter (2012) examined televised footage of individuals emotionally pleading to the public for the return of a missing relative, where half the pleaders were eventually convicted of killing the missing person while the other half were genuine pleaders. Microexpressions occurred only rarely and approximately equally across genuine and deceptive appeals. ten Brinke et al. (2011) examined microexpressions produced by participants expressing genuine or feigned remorse and reported that microexpressions occurred in less than 20% of the narratives studied and did not differentiate genuine from feigned remorse. Porter et al. (2012) examined whether the intensity of emotional arousal moderated the ability of microexpressions to differentiate truthful from deceptive individuals and reported that microexpressions could not do so.

These studies were important because they were the first examinations of the possibility that microexpressions could be indicators of deception.^1^ Although some findings provided tenuous support for the claim of microexpressions as indicators of deception (ten Brinke et al., 2011), generally the results indicated that microexpressions were not necessarily indicative of deception and that their rarity limited their potential as cues to deceit (although they may be signs of concealed emotions). Thus, findings to date regarding microexpressions as possible deception indicators have been equivocal at best, challenging popular notions.

#### Reconsidering the Operational Definition of Microexpressions

We introduce here and test below a methodological limitation about the operationalization of microexpressions in the studies to date. In the research described above, microexpressions were operationalized as expressions occurring between 1/25th and 1/5th s (i.e., 0.04–0.20 s), commensurate with previous claims concerning the duration of microexpressions (Ekman, 1985, 2009). A limitation concerning these and other claims about microexpression duration, however, is that a production study documenting the duration of microexpressions or their range had never been conducted.^2^ A production study documenting the speed of microexpressions would have had to have elicited emotions in individuals and measured not only the occurrence of the produced facial expressions of emotion but also their timing (onset, apex, and offset) and then empirically demonstrated that microexpressions were occurring at speeds as fast as 1/25th or 1/5th s. Such a study would also had to have demonstrated that the produced expressions were signs of concealed or suppressed emotions. Such a study has never been published; in a strict sense, therefore, previous claims about the speeds of microexpressions have been arbitrary (which may be one reason why claims about speeds have differed over the years).

More importantly for the purpose of this study, previous claims about the duration of microexpressions were not commensurate with previously published research documenting the duration of non-concealed, non-repressed, spontaneously occurring facial expressions of emotion as being between 0.67 and 4.0 s (Ekman et al., 1980; Ekman and Fridlund, 1987; Ekman et al., 1998; Frank et al., 1993). For example, when discussing the findings of one of the first studies to document the duration of normally occurring, spontaneously produced facial expressions of emotion (Ekman et al., 1980), Ekman and Friesen (1982) wrote:

“This experiment also suggested that there may be some boundaries to the usual duration of a felt smile. None were extremely brief nor were any very long. In other situations we also observed that most smiles were between two-thirds of a second and four seconds in length if they were felt.... When positive feelings are weak the smile involves only slight muscular contractions, which are infrequent and short, but rarely less than two-thirds of a second. When positive feelings are very high, the smile involves very strong muscular contractions, which happen often and are very long, but rarely more than four seconds.” (p. 244).

Later, Ekman and colleagues (Frank et al., 1993) revised the above duration description to 0.50–4.0 s for other emotional expressions as well (but the rationale for the change of the lower limit from 0.67 to 0.50 s was not clear).

Given this empirically documented duration range for non-concealed, non-repressed, spontaneously produced emotional expressions, our position is that defining microexpressions initially as expressions that occur more quickly than the lower limit of this empirically documented range makes the most sense and is empirically justified by the available research literature concerning expression duration. This suggests that an empirically justified operationalization of the upper limit duration range for microexpressions should be ≤0.50 s. We do so in this exploratory study and examine the occurrence of microexpressions systematically at lower durations as well (≤0.40, ≤0.30, and ≤0.20 s). This operationalization also implies that the operational definition of microexpressions in the previous studies described above (0.04–0.20 s) may have been too limiting (which may explain why they occurred so infrequently in those studies).

#### Other Conceptual Issues

Operationalizing microexpressions as expressions occurring ≤0.50 s raises other considerations. First, using a more liberal definition of microexpression duration, especially one that borders on the lower limit of naturally occurring, spontaneously produced, non-concealed expressions, raises questions about what phenomena the expressions reference. That is, given that previous claims about microexpressions suggested that they were signs of concealed or repressed emotions, defining microexpressions with longer durations may result in the identification of expressions that are no longer signs of concealed or repressed emotions. We acknowledge that possibility. In reality, however, as mentioned above there has never been a production study that has tested whether microexpressions of any duration occur (other than the fairly restrictive 0.04–0.20 s duration) or are indicators of deception. Given the lack of such evidence, our position is that the operationalization of microexpression duration used here is the cleanest methodological way to measure them in an initial, exploratory study. And, self-report data on subjective emotional experiences before and after the experiment are also reported below in order to approach understanding if emotional states were aroused.

An additional issue concerns the degree to which expressions at any speed are voluntary or involuntary. Our position is that the distinction between voluntary and involuntary expressions is not that clear and question whether that distinction can be made on the basis of expression duration. In fact empirical evidence does not exist that would suggest that an arbitrary cutoff based on expression duration exists that can delineate whether expressions are voluntary or not. On the one hand, as argued many years ago (Matsumoto and Lee, 1993), even normally occurring, spontaneously produced expressions are learned and practiced so well during the process of socialization and enculturation that they are naturally produced spontaneously but still outside of conscious awareness. To wit, individuals generally have little or no conscious awareness of the expressions they spontaneously produce (Barr and Kleck, 1995). Thus, many expressions produced spontaneously (and longer than 0.50 or 0.67 s in duration) are “unconscious” (with the obvious exception of simulated expressions produced at will).

On the other hand, extremely quick expressions are most likely produced involuntarily, probably because the neuropsychological wiring and processes do not lend to conscious, volitional thoughts to produce extremely quick expressions. The question, however, concerns if and whether there is a duration cutoff point for knowing exactly when these “completely” involuntary, extremely quick expressions that are signs of concealed emotions would overlap with involuntary yet spontaneously produced expressions that are not signs of concealed emotions. No one knows because no study to date has explored this issue.

Given this state of affairs we believe that the starting point for the first, exploratory production study to examine whether expressions of different durations occur and can differentiate truthtellers and liars should be ≤0.50 s, which is the empirically documented lower level duration of naturally occurring, spontaneously produced expressions of non-concealed emotions. This operationalization is the most logical given the available evidence to date (with systematically quicker durations also tested as done below).

### Overview of This Study

In this study we examined whether microexpressions ≤0.50 s occurred and if they were indicators of veracity and deception. Duration was defined as the total time from the onset of an expression through its apex and until its offset. As introduced above, because of the exploratory nature of the study, microexpressions were operationalized at various durations, starting at 0.04–0.20 s as this was that which was operationalized by the initial production studies concerning microexpressions reviewed above (Porter and ten Brinke, 2008; ten Brinke et al., 2011; Porter et al., 2012; ten Brinke and Porter, 2012). Microexpressions occurring ≤0.30, ≤0.40, and ≤0.50 s were also computed to examine different operationalizations of microexpressions occurring at or under the lower limit of spontaneously produced, non-concealed expressions (Ekman et al., 1980; Ekman and Friesen, 1982; Ekman and Fridlund, 1987; Frank et al., 1993; Ekman et al., 1998). Expressions between 0.50 and 6.00 s were also tested, as this range is the empirically verified range of normally occurring, spontaneous emotional expressions. These analyses allowed us to first examine if microexpressions at different speeds were produced and second if they differentiated truthtellers from liars.

Individuals participated in a mock crime experiment in which they had to either lie or tell the truth about a theft. Another unique aspect of this study was our focus on expressions produced by participants in an initial, checkpoint-type screening interview prior to their gaining access to an area where the theft could occur. Thus, this study examined the possibility of microexpressions to differentiate truthtellers from liars in their intent for a future malicious act as opposed to one that occurred in the past (the typical way in which deception studies have been conducted). The experimental context modeled actual real-life checkpoint security screening procedures in which actors with malicious intent need to hide their intentions and with stakes involved regarding whether or not they were believed. Because of that, participants should have had additional cognitive and emotional loads that they would have had to regulate, resulting in the possibility of emotional leakage and thus microexpressions.

This study also examined microexpressions produced by participants from two very different cultural/ethnic groups. Because facial expressions of emotion are universally produced and recognized (Hwang and Matsumoto, 2016), microexpressions should function similarly in different cultural/ethnic groups. We hypothesized that microexpressions, defined as those expressions occurring ≤0.50 s would occur and would differentiate truthtellers from liars.

## Materials and Methods^3^

### Design

The experiment was a two-way design with Veracity Condition (truths vs. lies) and Ethnicity (European Americans vs. Chinese immigrants) as factors. (Although we did not have hypotheses about sex differences, sex was included as a factor in the initial overall analyses below as well.) The experiment and record collection procedures have been previously described elsewhere, along with findings from other verbal and nonverbal behavior coded from a different interview in the same experiment (Matsumoto and Hwang, 2015, 2018; Matsumoto et al., 2015). The dependent variables in this article were facial expressions of emotions coded during the screening interview; these data were based on new manual coding of the archival video records of a different interview not previously done or reported elsewhere; therefore, the coding procedures, data, and findings reported here are entirely new to the literature.

### Participants

Participants were a community sample who responded to online ads and flyers recruiting individuals for a study examining cultural differences in how people feel when going through security interviews. The sample was comprised of two groups: *n* = 41 U.S. born-and-raised European Americans (*n* = 19 females, mean age = 26.22; *n* = 22 males, mean age = 23.68) and *n* = 36 Chinese immigrants (*n* = 19 females, mean age = 26.47; *n* = 17 males, mean age = 24.50); thus, the total sample size was *N* = 77. For the purposes of this study, Chinese immigrants were defined as individuals born and raised in the People’s Republic of China, Hong Kong, or Taiwan, or first generation born in the United States but whose first language was not English and whose parents were born in one of those countries. As explained below, participants were randomly assigned to either a truth or lie condition (*n*_truth_ = 37, *n*_lie_ = 40).

### Measures

Participants completed a demographics questionnaire, the General Ethnicity Questionnaire (GEQ; Tsai et al., 2000), the Machiavellianism Scale (Christie, 1970), and the Self-Monitoring Scale (Snyder, 1974). Participants also completed an emotion checklist at the beginning and end of the experiment. This checklist included 12 emotion words (guilt, fear, anger, embarrassment, worry, contempt, excitement, disgust, amusement, nervousness, surprise, and interest) rated on nine-point scales labeled 0 = *None*, 4 = *Moderate Amount*, and 8 = *Extremely Strong*. All measures except the emotion checklist were omitted from this study.

The GEQ is a commonly used measure of acculturation and ethnic identity and was included as a manipulation check for ethnic/cultural differences. It contained 38 statements, 25 rated on a 5-point Likert scale from Strongly Disagree to Strongly Agree, and 13 rated on a 5-point scale from Very Much to Not at All. The target ethnicity in the GEQ was modified to be Chinese. The GEQ Total score, which was the mean of all items after reverse coding those negatively loaded, indicated that the Chinese sample had significantly higher scores than American born Chinese and Chinese who immigrated before the age of 12 years as reported by Tsai et al. (2000), *t*(35) = 10.16, *p* < 0.001, *d* = 1.72; and *t*(35) = 4.50, *p* < 0.001, *d* = 0.76, respectively. These analyses demonstrated that our Chinese sample identified themselves as Chinese and strongly with Chinese culture and more so than American born Chinese.

### Procedures

After consenting, participants first completed the pre-session measures, after which they were told that they would be randomly assigned to either take a $100 check made out to cash or to look at but not take the $100 check. They were told that their goal was to go through up to three checkpoints/interviews and that they needed to convince all officers of their honesty, sincerity, and innocence. Participants were informed of the stakes involved: that they will earn a minimum of $20 for their participation and bonuses of anywhere from $0 to $80 depending upon the determinations of the interviewers. If they were believed by all interviewers, they received additional money and were allowed to leave early; but if they were not believed by any one interviewer, they received no additional money and had to stay an additional hour completing a long questionnaire. After confirming their understanding of the instructions and stakes, participants rated the severity of the expected consequences if they were judged to be lying in the experiment using a scale from 0, No consequence, to 10, Maximum consequence. The mean was 5.75 (*SD* = 1.83), which was significantly greater than zero, *t*(74) = 27.18, *p* < 0.001, *d* = 3.14, and suggested that the participants perceived the stakes on a moderate level. A random assignment procedure was then conducted in front of the participant at which time they learned their assigned condition.

Participants were then escorted out of the instruction room to a separate area and staged in an area modeled after a screening checkpoint. After waiting a few minutes alone, the initial screening interview occurred. Six male actors, some of whom were former law enforcement officers and all above the age of 30 years, served as interviewers. All interviewers were trained to deliver the interviews in a neutral and objective manner and to stick with the predetermined interview questions. The questions were modeled after those used in real-life security checkpoints and developed after consultation with Subject Matter Experts (SMEs) from various law enforcement entities with interests in the practical application of the findings. Thus, the questions were designed to be as realistic as possible yet to retain fidelity for research purposes.

An interviewer wearing a standard security uniform appeared, walked by the participant, stepped behind a checkpoint interview table (podium), and told the participant to empty their pockets before going through a metal detector. The interviewer then conducted the screening interview, which included seven questions and lasted an average of 1:56 m. When the screening interview was done, the interviewer left and the remainder of the experiment occurred, including a secondary interview, the mock crime, and an investigative interview, after which post-session measures were administered followed by debriefing, post-session consent, and payment. Because the focus of this study was on the initial screening interview, no further mention of the remainder of the experiment will be made.

### Analysis of Facial Expressive Behavior

Facial behaviors that spontaneously occurred during the initial screening interview were coded by two facial coding experts (one with 35+ years of experience, the other with 10+ years of experience) using a modified version of the Facial Action Coding System (FACS; Ekman and Friesen, 1978) known as Emotion FACS (EMFACS; Matsumoto et al., 1991). Both coders were blind to the veracity condition of the participants. EMFACS is an abbreviated version of FACS that identifies validated facial behaviors associated with known emotional states based on previous theory and research (Hwang and Matsumoto, 2016). EMFACS coding was done in real-time and identified the occurrence of seven emotions: anger, contempt, disgust, fear, happiness, sadness, or surprise. Onset and offset times of each expression were also denoted, allowing for computation of expression durations. Reliabilities were acceptable for all emotions separately (% agreement = 0.70, 0.91, 0.75, 0.96, 0.67, 0.68, and 0.82 for anger, contempt, disgust, fear, happiness, sadness, and surprise, respectively) and for the total emotions coded, *r*(76) = 0.97. All codes used for analyses below were those in which both coders agreed.

### Ratings of Interviewer Contamination

Interviewers can impede or negatively influence the interview process, thereby influencing participant emotions and expressions and possibly obscuring the clarity of the data obtained. For example, sometimes an interviewer can misstate or rearrange words of a question, altering its meaning; interrupt a participant when he or she was responding; interject words or phrases during a participant’s response such as, “*keep going*,” “*go on*”; or volunteer words to help a participant complete a response. In order to assess the degree to which interviewer contamination may have influenced our data, two coders with 20+ years of investigative experience in law enforcement coded the transcripts from all interviews for interviewer contamination. Reliability between the coders was 0.83. Contamination occurred minimally in the initial screening interview. Nevertheless, below we present results filtered for interviewer contamination, which accounts for differences in *df*s.

## Results

### Manipulation Checks

In addition to the ratings of the stakes involved and the GEQ described above, we examined whether participants were emotionally aroused during the experiment by conducting a five-way, mixed ANOVA on the emotion ratings using Ethnicity (2), Sex (2), and Veracity Condition (2) as between subject factors and Pre–Post (2) and Emotion (12) as within subject factors. The Pre-Post by Emotion by Veracity Condition interaction was significant, *F*(11,682) = 3.46, *p* < 0.001, $\eta_{p}^{2}$= 0.053. No interaction involving Ethnicity or Sex was significant. We decomposed the significant three-way by computing simple effects of Pre–Post separately for each emotion and veracity condition. Liars *increased* in guilt, fear, embarrassment, contempt, disgust, and amusement while truthtellers *decreased* in guilt, fear, embarrassment, worry, excitement, and increased in amusement (Cohen’s *d* = -0.75, -0.32, -0.40, -0.37, -0.22, -0.45, and 0.77, 0.40, 0.36, 0.80, 0.38, and -0.50, respectively). Thus, the participants, especially the liars, were emotionally aroused during the procedures.

### Preliminary Analyses

Although the initial screening interview contained seven questions, our analysis focused on three questions to which participants assigned to the steal-lie condition had to lie but truthtellers could simply tell the truth, consistent with the deception literature. We first computed descriptive statistics (Ms and SDs) for the occurrence of each of the seven emotions coded and did so at the varying expression durations described above to examine different operationalizations of microexpressions. As shown in Table 1, expressions occurred very rarely at 0.04–0.20 s and even at ≤0.30 s. But they did occur more frequently from ≤0.40 s and higher. The lack of microexpressions at 0.04–0.20 s was commensurate with previous findings.

**Table 1:** Descriptive statistics (means and standard deviations) of the occurrence of facial expressions of emotion separately by veracity condition and duration.

Duration	Condition	Anger	Contempt	Disgust	Fear	Happiness	Sadness	Surprise
0.04–0.20 s	Truth	0.00 (0.00)	0.00 (0.00)	0.00 (0.00)	0.00 (0.00)	0.00 (0.00)	0.00 (0.00)	0.00 (0.00)
	Lie	0.00 (0.00)	0.03 (0.16)	0.00 (0.00)	0.00 (0.00)	0.00 (0.00)	0.00 (0.00)	0.00 (0.00)
≤0.30 s	Truth	0.00 (0.00)	0.00 (0.00)	0.00 (0.00)	0.00 (0.00)	0.00 (0.00)	0.00 (0.00)	0.00 (0.00)
	Lie	0.00 (0.00)	0.03 (0.16)	0.00 (0.00)	0.08 (0.27)	0.00 (0.00)	0.03 (0.16)	0.00 (0.00)
≤0.40 s	Truth	0.00 (0.00)	0.00 (0.00)	0.00 (0.00)	0.00 (0.00)	0.00 (0.00)	0.00 (0.00)	0.00 (0.00)
	Lie	0.08 (0.27)	0.05 (0.22)	0.08 (0.27)	0.13 (0.40)	0.00 (0.00)	0.03 (0.16)	0.00 (0.00)
≤0.50 s	Truth	0.00 (0.00)	0.00 (0.00)	0.03 (0.17)	0.03 (0.17)	0.00 (0.00)	0.03 (0.17)	0.00 (0.00)
	Lie	0.10 (0.30)	0.23 (0.66)	0.10 (0.30)	0.23 (0.66)	0.00 (0.00)	0.10 (0.30)	0.00 (0.00)
0.50–6.0 s	Truth	0.03 (0.17)	0.11 (0.52)	0.00 (0.00)	0.22 (0.42)	0.11 (0.40)	0.14 (0.35)	0.11 (0.32)
	Lie	0.18 (0.59)	0.33 (0.69)	0.13 (0.65)	0.62 (0.87)	0.05 (0.32)	0.38 (0.70)	0.03 (0.16)
≤6.00 s	Truth	0.03 (0.17)	0.11 (0.52)	0.03 (0.17)	0.25 (0.44)	0.11 (0.40)	0.17 (0.38)	0.11 (0.32)
	Lie	0.25 (0.63)	0.53 (1.04)	0.20 (0.72)	0.85 (1.14)	0.05 (0.32)	0.48 (0.85)	0.03 (0.16)

### Main Analyses

Because of low frequencies of anger, contempt, disgust, fear, and sad expressions, these were combined into a total negative (NEG) category for each duration. We then computed separate, three-way univariate ANOVAs on the NEG and happy (HA) expressions using Veracity Condition (2), Ethnicity (2), and Participant Sex (2) as factors, separately for each duration. No significant effects were produced for happy expressions at any expression duration. Also, there were no significant effects produced at 0.04–0.20 s duration, replicating the non-findings reported previously at this duration (Porter and ten Brinke, 2008; ten Brinke et al., 2011; Porter et al., 2012; ten Brinke and Porter, 2012). The Veracity condition main effect was also not significant for expressions ≤0.30 s.

Significant Veracity condition main effects were produced, however, at each of the other durations, indicating that liars produced more negative expressions than did truthtellers. The fastest speed at which this differentiation occurred was ≤0.40 s. Effect size estimates ($\eta_{p}^{2}$ and Cohen’s *d*) for these effects were substantial and the upper and lower level 95% CIs of the means did not overlap between truthtellers and liars. These statistics provided strong support for microexpressions ≤0.40 and ≤0.50 s to differentiate truthtellers and liars, as well as for longer expressions (0.50–6.00 s) to do so (see Table 2 for summary of significant Veracity condition main effects).

**Table 2:** Summary of veracity condition main effects produced by univariate ANOVAs examining veracity condition, ethnicity, and participant sex on combined negative (NEG) expressions at different durations.

Expression duration	Veracity condition	*M* (*SD*)	LLCI	ULCI	*F*	*df*	*p*	$\eta_{p}^{2}$	Cohen’s *d*
0.04–0.20 s	Truth	0.00 (0.00)	-0.04	0.04	1.79	1,68	0.185	0.026	0.38
	Lie	0.03 (0.16)	-0.001	0.07					
≤0.30 s	Truth	0.00 (0.00)	-0.10	0.10	2.38	1,68	0.127	0.034	0.65
	Lie	0.13 (0.40)	0.01	0.21					
≤0.40 s	Truth	0.00 (0.00)	-0.14	0.14	9.04	1,68	0.004	0.117	1.21
	Lie	0.35 (0.58)	0.16	0.44					
≤0.50 s	Truth	0.08 (0.28)	-0.17	0.35	9.54	1,68	0.003	0.123	1.02
	Lie	0.75 (1.03)	0.40	0.91					
0.50–6.0 s	Truth	0.50 (0.74)	0.18	0.88	20.94	1,68	<0.001	0.235	1.15
	Lie	1.64 (1.21)	1.31	1.99					
≤6.00 s	Truth	0.58 (0.84)	0.19	1.05	28.38	1,68	<0.001	0.294	1.43
	Lie	2.30 (1.56)	1.81	2.65					

To explore these effects further, we classified participants according to whether or not they produced *any* negative expressions, cross-tabulated this classification against Veracity Condition, and then computed chi-squares and Contingency Coefficients for each cross-tabulation, separately for each duration (Table 3). Once again, there was no difference between the percentage of truthtellers and liars producing negative expressions at 0.04–0.20 or ≤0.30 s. But there were significant differences in this percentage for all other durations, indicating that liars were significantly more likely than truthtellers to produce at least one negative expression during these durations. Correct classification rates were 63.2% and 68.4% for microexpressions ≤0.40 and ≤0.50 s, respectively, which were higher than the rates for lay observers (Bond and DePaulo, 2006).

**Table 3:** Cross-tabulations, chi-squares, and contingency coefficients for participants who produced any negative expressions as a function of veracity condition.

Expression duration	Veracity condition	None (%)	One or more (%)	Total % correctly classified	χ^2^	*p*	CC
**Number of negative expressions**							
0.04–0.20 s	Truth	47.4	0.0	48.7	χ^2^(76) = 0.91	0.340	0.11
	Lie	51.3	1.3				
≤0.30 s	Truth	47.4	0.0	52.7	χ^2^(76) = 3.80	0.051	0.22
	Lie	47.4	5.3				
≤0.40 s	Truth	47.4	0.0	63.2	χ^2^(76) = 12.83	<0.001	0.38
	Lie	36.8	15.8				
<0.50 s	Truth	43.4	3.9	68.4	χ^2^(76) = 14.13	<0.001	0.40
	Lie	27.6	25.0				
0.50–6.0 s	Truth	28.9	18.4	72.3	χ^2^(76) = 15.27	<0.001	0.41
	Lie	9.2	43.4				
≤6.00 s	Truth	27.6	19.7	75.0	χ^2^(76) = 20.05	<0.001	0.46
	Lie	5.3	47.4				
***Post hoc* analyses**							
<1.00 s	Truth	34.2	13.2	77.6	χ^2^(76) = 23.10	<0.001	0.48
	Lie	9.2	43.4				
1.00–6.00 s	Truth	38.2	9.2	57.9	χ^2^(76) = 3.00	0.083	0.20
	Lie	32.9	19.7				

### *Post Hoc* Analyses

The analyses above also indicated that all expressions (i.e., ≤6.00 s) differentiated truthtellers from liars (Tables 2, 3). *Post hoc* cross-tabulation analyses, however, indicated that expressions ≤1.00 s drove this effect with a 77.6% correct classification rate. Filtering expressions ≤1.00 s from all expressions indicated that expressions that occurred between 1.00 and 6.00 s actually did not significantly differentiate truthtellers from liars (see Table 3, bottom).

**FIGURE 1:**
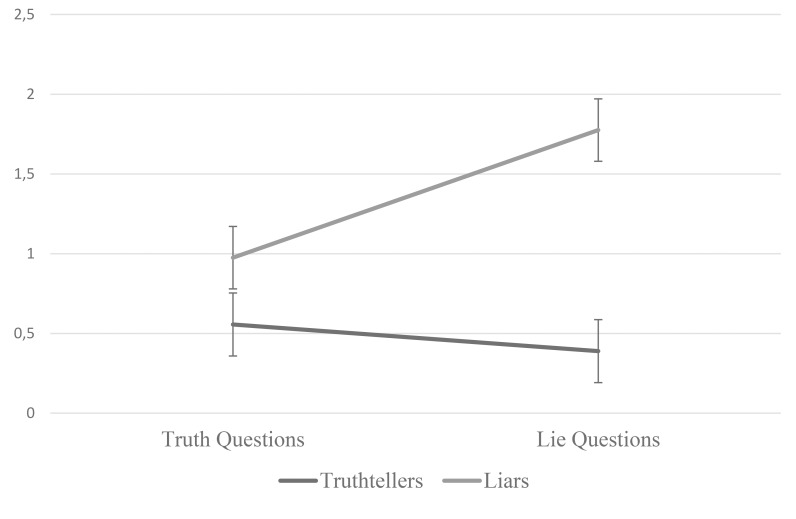
Changes in combined negative expressions by question type and veracity condition. NB, error bars are standard errors.

The above analyses focused on only the questions to which liars had to lie while truthtellers could just tell the truth, which was consistent with the literature. But the participants’ emotional expressions to other questions were coded as well. To explore the possibility that expressions of negative emotions increased across truth–lie questions for liars, we computed a two-way, mixed factorial ANOVA on the combined negative expression scores using Question Type as a within subjects variable and Veracity Condition as between. This analysis was done using expressions ≤1.00 s but the same effects were found with expressions ≤0.50 s. The interaction was significant, *F*(1,74) = 6.78, *p* = 0.011, $\eta_{p}^{2}$ = 0.084 (Figure 1); simple effects analyses indicated that negative expressions significantly increased for liars but not for truthtellers, *F*(1,39) = 6.00, *p* = 0.019, $\eta_{p}^{2}$ = 0.133; and *F*(1,35) = 1.30, *p* = 0.263, $\eta_{p}^{2}$ = 0.036, respectively. To our knowledge this is the first evidence in the literature to test changes in expressions for truthtellers and liars against a baseline.

## Discussion

The findings provided the first empirical support for the notion that microexpressions ≤0.40 and ≤0.50 s occur with enough frequency to differentiate truthtellers from liars. Interestingly, microexpressions occurring ≤0.30 did not do so. While all expressions (≤6.00 s) differentiated truthtellers from liars, this effect did not survive when expressions <1.00 s were filtered from the analyses; expressions ≤1.00 s differentiated truthtellers from liars with the highest accuracy classification. Also, microexpressions operationalized restrictively as those expressions that occurred between 1/25th and 1/5th s (0.04–0.20 s) occurred very rarely and did not differentiate truthtellers and liars.

This study was not conducted without limitations, perhaps the largest of which was the lack of adequate sample sizes to test interactions involving ethnicity or sex, precluding more statistically powerful tests of culture/ethnicity or gender moderation of the findings. Future research should address this limitation with additional and larger samples, not only to replicate the main findings but to better test these and other possible moderators. Also, the study involved only one type of lie (about future malicious intent) in one type of context (security checkpoint screening interview). Clearly, the potential for microexpressions to differentiate truthtellers and liars in other types of lies and contexts need to be examined in the future.

Another limitation of the study was the use of composite negative scores. Elsewhere we have provided a conceptual framework with which to understand and predict individual differences in emotional reactions when lying that would suggest the differential production of negative emotions when lying (Matsumoto and Hwang, 2018). But to date there has been no research explicating the individual difference variables that are associated with which emotions will be experienced or expressed by which individuals. Lacking such evidence and given the exploratory nature of this initial production study and the sample size available to analyze in this study, we deemed the computation and use of composite negative scores as reasonable. The total sample size allowed us to address the main purpose of the study – to test for the existence and duration of microexpressions and to examine if they differentiated truthtellers and liars – but would be unacceptable to test emotion-specific hypotheses. Also, the use of composite scores has the potential to inflate true differences between truthtellers and liars, especially related to smaller sample sizes, which is a common issue in deception research (for a review and excellent discussion on this topic, see Luke, 2018, August 6). Our findings should be interpreted in light of this strong caveat and should be replicated in a different study with larger sample sizes and pre-registering of hypotheses and analytic choices. The wide distribution of various types of negative expressions displayed by liars certainly suggested large individual differences in reactions to lying and responses to context.

Regardless, the current findings have several important ramifications. Despite years of claims that microexpressions are reliable indicators of deception, the findings reported here are the first in the scientific literature to provide evidence for their occurrence at different durations and for that claim. Microexpressions were operationalized on the basis of existing scientific evidence concerning the durations of normal, spontaneously occurring, non-concealed, non-repressed emotional expressions. Given this empirical evidence, we operationalized microexpressions as expressions lasting ≤0.20, ≤0.30, ≤0.40, and ≤0.50 s. We also tested expressions occurring during the durations of normal, spontaneously occurring, non-concealed, non-repressed emotional expressions (0.50–6.00 s). Positive findings occurred starting at ≤0.40 s.

Expressions that occurred ≤6.00 s also produced positive findings, suggesting that macroexpressions could also differentiate truthtellers from liars. But this effect did not survive when microexpressions ≤1.00 s were filtered from the analyses, suggesting that negative expressions that occurred within the normal ranges of spontaneously produced expressions (i.e., not micros) were not as reliable indicators of deception (allowing for overlap of micro- and macro-expressions between 0.50 and 1.00 s). This may have occurred because even truthtellers can and will experience negative emotions when being interrogated and their intentions questioned. To wit, note the non-insubstantial percentages of truthtellers who expressed some negative feelings in Table 3.

The data in Table 1 suggested large individual differences in the types of negative emotions participants experienced; while some got angry, others were disgusted, and yet others were afraid or sad. But the difference between truthtellers’ and liars’ expressions of negative emotions may be in their concealment; liars were more likely to hide or suppress their negative feelings, resulting in more microexpressions. Truthtellers, however, were less likely to do so, resulting in more normally appearing expressions (i.e., expressions with longer durations). As mentioned above, these findings open the door to future studies exploring individual differences in emotional reactivity in investigative interview contexts.

The weaker findings on facial expressions of emotion at longer durations may also give hints as to why previous studies and reviews examining facial expressions of emotion as possible deception indicators failed to provide reliable evidence for that notion. Close examination of the studies examining facial expressions in a review of the literature examining nonverbal clues to deception (DePaulo et al., 2003) indicates that no study examining facial expressions had conducted detailed facial measurement and assessed expressions at different durations as the current study did. The findings here, therefore, encourage closer, more precise examinations of the possibility that facial expressions of emotion, and microexpressions in particular, are indicators of deception.

The above findings were also noteworthy because of the type of lie examined. Most deception studies to date have examined lies concerning an incident in the past. In contrast, the current study examined lies about the intent to commit a malicious act in the future. Theoretically, such lies are different than lies about the past because they access different domains of cognition and memory. Fortunately, there is now a burgeoning literature examining differences between true and false intent (Granhag and Mac Giolla, 2014); but only a few studies of lies about intent for future malfeasance in criminal contexts exist (Burgoon et al., 2009; Vrij et al., 2011; Matsumoto et al., 2015). The current study adds to this small but growing literature. The results of this study and future studies on this topic can be helpful in informing practitioners about the validated behavioral indicators that occur in brief, checkpoint-type interactions and would have implications to security procedures in a wide variety of settings that assess future malicious intent.

That the findings were not moderated by the ethnicity or sex of the participants was also noteworthy (but note the sample size limitation to test moderation effects described above). In particular, the results of the GEQ documented the fact that the Chinese immigrant sample was substantially culturally different than the U.S. born-and-raised European American sample. In fact there are many differences between Chinese and Chinese immigrant cultures and mainstream European American cultures in cognitions, preferred emotions and emotion-related attitudes and values, and belief systems (e.g., see Tsai et al., 2000, 2002, 2006; Wang, 2006; Wang and Senzaki, 2017). Because of these cultural differences, the current findings lend credence to the utility and function of facial expressions of emotion in general, and microexpressions in particular, across cultures, consistent with the wealth of evidence documenting the universality of facial expressions of emotion (Hwang and Matsumoto, 2016). Future studies should include individuals from other cultural groups.

The findings reported here have several implications. Theoretically, they suggest a reconsideration of the role of facial expressions of emotion in general, and microexpressions in particular, vis-à-vis veracity and deception. Given the transient nature of emotion and emotional expressions and the dynamic nature of any interaction, microexpressions may play a different role as indicators of mental states than normally occurring, spontaneously produced facial expressions. Additionally, the type of lie being committed and the types of questions being asked and answered likely moderate the function of emotional expressions as will the behaviors and demeanor of the interviewer. All these factors need to be accounted for in a more comprehensive framework in the future.

Our operationalization of microexpressions based on expression durations using the empirically documented lower limit of normally occurring, spontaneously produced facial expressions of emotion (≤0.50 s) raises conceptual questions concerning whether these expressions are involuntary signs of concealed emotions. As discussed in the section “Introduction,” however, the voluntary–involuntary distinction vis-à-vis facial expressions of emotion is quite difficult to explicate because normally occurring, spontaneously produced emotional expressions at longer durations (0.50–6.00 s) can be and often are produced outside of conscious awareness. Moreover, measuring consciousness and volition with regard to expression production is very difficult because most people are not aware of what their faces are doing even when they are not lying (Barr and Kleck, 1995). Measuring the concealment of emotion would also be extremely difficult given that the association between expression and self-report is transient (Rosenberg and Ekman, 1994) and many emotional states are themselves unconscious. For these reasons we make no interpretation concerning whether or not expressions ≤0.50 s are conscious or unconscious, volitional or involitional, as we believe these distinctions are virtually impossible to assess.

Also, we offer our operationalization of microexpressions using expression duration as a start, not end, for discussions, argument, disagreement, and research on these issues. Given the ambiguity of consciousness, volition, and concealment, we deemed the use of expression duration as the cleanest way with which to measure microexpressions. Other researchers may or may not agree with our starting point. We hope that the transparency about our operationalization and the exploratory nature of this study allows for further research and theory on previously unfounded (but interesting) claims. We certainly invite that further work on this topic.

Empirically, the current findings suggest that future studies examining the possible role of facial expressions of emotion and microexpressions utilize direct and detailed facial measurement and test expressions at different expression durations in order to investigate whether they occur and differentiate truths from lies. In particular, future studies need to replicate the findings reported here, hopefully with larger sample sizes that can allow for the testing of single emotions (instead of composite scores) with preregistered hypotheses. The current study opens the door to such studies conducted carefully and with methodological rigor concerning the operationalization of microexpressions.

Finally, the current findings have applied implications. Checkpoints and screening interviews are important layers of security in many domains of daily life, including airports, military installations, government facilities, private businesses, public venues, and the like. These areas may be enhanced by security professionals being trained to identify and interpret microexpressions accurately within a conversational or interview protocol. To be sure, there are always reasonable questions concerning the generalizability of findings from controlled experiments such as this one to actual, real-life security contexts, and individuals interested in application should raise such questions. Real life security contexts are likely to arouse emotions in actors even more strongly than in a controlled experiment, thus leading to greater possibility of microexpressions and other behavioral indicators of intent and deception. Individuals can be trained to spot microexpressions (Matsumoto and Hwang, 2011) and computer science technologies are being developed that can better identify nonverbal behavior and may play a role in such security contexts (Perez-Rosas et al., 2015; Carissimi et al., 2018). Placed carefully and strategically within a layered security approach, the identification of microexpressions can substantially enhance security operations for all such venues.

## Ethics Statement

The study reported in this article involved the coding of already-collected, archival video records and was thus exempted from Human Subjects review.

## Author Contributions

Both authors designed the study, supervised data collection, coded the source video records, and contributed equally to the reviewing and editing of the drafts to final. DM conducted the preliminary data analyses and wrote the first draft of the manuscript and HH conducted the confirmation analyses.

## Conflict of Interest Statement

The authors are employees of Humintell, a for-profit entity that sells microexpression-related products.

## Footnotes

1 Zurloni et al. (2015) also examined microexpressions and other nonverbal behavior in a case study of the expressive behavior of Lance Armstrong to differentiate truthful and deceptive statements. No specific operationalization of microexpressions was described, however, especially concerning expression duration.

2 Additionally questions concerning the rationale underlying claims concerning the occurrence of microexpressions as brief as 1/25th s exist. The film records of depressive inpatients that were the basis for Ekman and Friesen’s (1969, 1974) original discussion about micros were recorded at 24 frames per second (fps). Later records used video with recording speeds of 30 fps. In either case, documenting micros at 1/25th s is quite unlikely given these characteristics of the available source records. To be sure, specialized video cameras can record at much higher fps, such as 60 fps, 200 fps, or even more, which we have used in past research. But no documentation of such recording or microexpression duration has ever been noted, and in any case as mentioned above, no published study has ever documented the existence of micros or their characteristic as indicators of deception, as quality control required through the review and publication process would likely have required documentation of the durations of the expressions analyzed.

3 The collection of the original archival video records were approved by the University at Buffalo, State University of New York Social and Behavioral Sciences Institutional Review Board. Written consent was obtained from all interviewees appearing in the video records after they were fully informed about the experimental procedures.
